# A data portrait-based approach to precision education for medical students in China

**DOI:** 10.1097/MD.0000000000047664

**Published:** 2026-02-13

**Authors:** Shaojie Yu, Xuehong Ju, Chunguang Ling

**Affiliations:** a School of Psychology, Shandong Second Medical University, Weifang, China; b School of Graduate, Shandong Second Medical University, Weifang, China.

**Keywords:** data portrait, medical students, precision education

## Abstract

With the rapid development of big data technology, smart campuses from medical universities of China have accumulated a large amount of student behavior data. How to extract hidden and valuable information from a large amount of data has become a problem faced by medical universities of China. This study constructed a framework for the portrait model of medical university students of China, which includes 5 dimensions: identity and organizational data, learning data, economic data, and health and interest data. These data are sourced from the smart campus information system of medical universities of China. Then, *K*-means clustering method is used to calculate these data, extract the characteristic attributes of students, and construct accurate student data portraits. This study uses big data profiling technology to accurately describe the digital characteristics of students, providing reference for the educational environment of medical universities.

## 1. Introduction

Driven by the digitization of education, as an important symbol of modernization in higher education, smart campus of China is reshaping the ecological pattern of higher education at an unprecedented speed. With the continuous deepening of big data technology applications, the construction of smart campuses in medical universities is becoming increasingly perfect. Smart campus can collect a large amount of multidimensional and real-time behavioral data of university students, forming a huge and complex database. These data not only bring unprecedented challenges to university management, but also nurture unprecedented opportunities. By deeply mining these data, information can be transformed into value, providing solid data support for university decision-making.^[1]^ In this context, data portrait has become the core issue and key breakthrough point in smart campus data mining. Data portrait is a bridge connecting the data world and the real world. Its essence is to comprehensively integrate, deeply mine, and accurately depict university student data scattered in various modules of the smart campus through data processing and analysis techniques, thereby constructing a multidimensional, multi-level, and labeled student feature model.^[2]^ The student profiles in medical universities not only involves the collection and analysis of explicit data such as basic information, learning behavior, life trajectory, and interests, but also delves into implicit characteristics such as students’ psychological state, values, and social networks, striving to comprehensively and accurately construct a digital portrait of each student.

Accurately profiling students through data has profound significance for the innovation and development of precision education in medical universities. It breaks the limitations of traditional methods that rely on empirical judgment and subjective speculation, enabling teachers to accurately grasp students’ ideological dynamics, behavioral patterns, and growth needs based on objective data, and implement more personalized, differentiated, and accurate educational methods.^[3]^ This approach provides quantitative indicators and scientific basis for evaluating educational effectiveness, helping teachers adjust their education plans, optimize the allocation of educational resources, and enhance the effectiveness of precision education. Data analysis is not only a practice of big data technology, but also a way to promote the scientific, intelligent, and precise development. Data profiling has unique advantages, providing new perspectives and tools for teachers to tackle challenges in complex environments.

## 2. Literature review

The research on data portrait focuses on several fields such as concepts, characteristics, data collection and processing. Portrait is the virtual representation of user information in real life, and is a target user model based on a large amount of real user data.^[4]^ Product users play an important role in the product development process. User profiles can describe the target audience for selling products. User profiles are models generated by precise descriptions by users, and their characteristics include photos, names, interests, hobbies, etc.^[5]^ The data portrait is a labeled user profiling model that incorporates demographic indicators such as user gender, education level, social interaction situation, and personal behavior patterns, and then analyzes, summarizes, and concludes them.^[6]^ It puts user experience in an important position. The data portrait has characteristics such as fundamental, authenticity, targetedness, uniqueness, and empathy.^[7]^ Under the background of data, portrait has the characteristics of iterability, timeliness, distinguishability, interactivity, knowledge, and clustering.^[8]^ Data portrait is a new research field, but the research results are rich, laying the foundation for subsequent theoretical research and its application. However, we should be noted that current research on data portrait at home and abroad needs to be further strengthened, such as the lack of a complete and mature theory and system, the construction process of portrait models, the accuracy judgment of portrait results, the evaluation and feedback system of portrait models, etc.

The data portrait has a wide range of application fields, covering e-commerce trade activities, smart tourism, library construction and services, and big data health and healthcare. In the field of e-commerce, data portrait is mainly applied in personalized product recommendation and service information push based on customer characteristics.^[9]^ In the field of smart tourism, collaborative filtering technology is used to predict potential effective information of users based on their browsing history, and high-quality tourism information is timely pushed to users. Potential users can also be discovered through data association technology.^[10]^ In the field of healthcare, elderly chronic disease users have constructed accurate user profiles through data generation, storage, and interaction, and developed intelligent healthcare systems and medical health apps targeting elderly chronic disease users based on their specific needs and characteristics.^[11]^ The above research provides experience for the application of data portrait in precise education of medical university students. In the internet era, where the amount of data is increasing, privacy protection is more challenging. All parties need to make efforts in policies, laws, ethics, user information literacy education and other aspects. Governments around the world are continuously strengthening their emphasis on personal privacy protection. In 2018, the European Union passed the “General Data Protection Regulations”. In 2021, China introduced the “Personal Information Protection Law”.

The combination of data portrait and precise education for medical university students is not only a development trend precise education, but also a requirement for data portrait. Traditional education research is mostly observational, and it is partial and deeply constrained by small sample data even with data support. Precise education supported by big data is based on big data, achieving personalized and diversified aggregation, transformation, extraction, and expression of data. Life habits, learning characteristics, and even ideological dynamics can be measured through big data, which provides accurate and visual tools for precise education in medical university.^[12]^ Data has the characteristics of diverse subjects, diverse content, and diverse fields, providing unlimited possibilities for precise education of medical university. Accurately identifying big data can locate teachers’ work tasks, determine students’ positions in the group, and achieve precise matching of education.^[13]^ Cloud computing, big data, artificial intelligence, and blockchain are the foundations of data portrait. The data portrait has constructed a “knowledge, emotion, intention, and action” map for precision education of medical university students, which is conducive to understanding and approaching students.^[14]^ The data portrait has matured and can provide technical support for precise education for medical university students. However, there are still 2 issues in the specific application of data portrait: the data portrait faces external challenges, such as dilution of information attention of educational objects, digital dependence of teacher, data information security and ethical crisis; The data portrait faces internal pressure, such as the lack of precise education concepts, imperfect coordination mechanisms for precise education, and a lack of data professionals.

The precise education model consists of a demand module, a data layer library module, and a portrait visualization module.^[15]^ In China, some universities have already integrated big data profiling and precise ideological and political education into educational practice. The University of Electronic Science and Technology of China fully relies on its technological advantages to continuously promote the deepening of ideological and political education work, truly implementing a high degree of integration between ideological and political work and information technology. The Education Department of Sichuan Province of China has been promoting the implementation of “precision ideological and political education” since 2020. The learning status, behavior, and results of over 1.5 million university students of Sichuan Province have been visualized, quantitatively measured, and summarized. Through the big data profiling, universities of Sichuan Province can obtain a map of university students’ knowledge, emotions, intentions, and actions, and use it accurately throughout the entire process of ideological and political education.^[16]^ The 2020 Symposium on Innovative Development of Ideological and Political Work was held at Shanghai Jiao Tong University in August 2020. Shanghai Jiao Tong University and University of Electronic Science and Technology of China introduced their experiences at the conference. The integration of traditional advantages of ideological and political work with modern information technology is an inevitable trend in the development of innovative ideological and political work, a practical need to solve the problems of ideological and political work, and an important measure to empower precision ideological and political work and enhance its effectiveness^.[17]^ Shandong University of Technology divides the course of “Marxist Principles” into 3 stages: pre-course, in-course, and post-course. Teachers provide precise portraits of students, provide precise teaching design, and effectively improve teaching effectiveness.^[18]^

## 3. Methods

### 3.1. Dimensions of student characteristics

This study extracts and mines data on the comprehensive behavioral characteristics of students. Based on the time and spatial distribution of students’ learning and life, the profile is drawn from 5 dimensions: identity data, learning data, economic data, health data, and interest data. The detailed content of each dimension is shown in Table 1. Identity data is a static feature attribute, including name data, gender data, age data, grade data, major data, etc, these data is obtained from the Student System. Learning data refers to the course and grade information of students, including course data, grade data, subject competition data, etc, which are obtained from Teaching System. Economic data refers to the economic income and consumption information of students, including scholarship data, work study income data, research assistant subsidy data, meal cost data, and on campus shopping data, these data is obtained from Student System, Logistics System, and Academic Research System. Health data refers to the physical and mental health information of students, including basic disease data, physical education class performance data, physical exercise data, school hospital medical data, psychological counseling data, etc, these data is obtained from the Student System, Teaching System, University Hospital System, and Psychological Counseling System; the interest data is the basis for the dimensional classification of students, and it is necessary to combine feature attributes to better describe the overall situation of student portraits, these data is obtained from the Library System, Network System, Student System, and Student Quality Expansion System.

**Table 1:** The dimensions of student portraits.

Dimensions	Content	Data sources
Identity	Name, gender, age, province, college, grade, major	Student System
Learning	Curriculums, grades, subject competitions	Teaching System
Economic	Scholarships, work study programs, research assistant subsidies, meal vouchers, shopping expenses	Student System, Logistics System, and Research System
Health	Basic diseases, physical education grades, physical exercise, treatment at the school hospital, psychological counseling	Student System,Teaching System, University Hospital System, Psychological Counseling System
Interest	Book borrowing, internet access, volunteer services, and appointment at the Student Quality Development Center	Library system, Network System, Student System, Student Quality Expansion System

### 3.2. Student portrait framework

**Figure 1.**
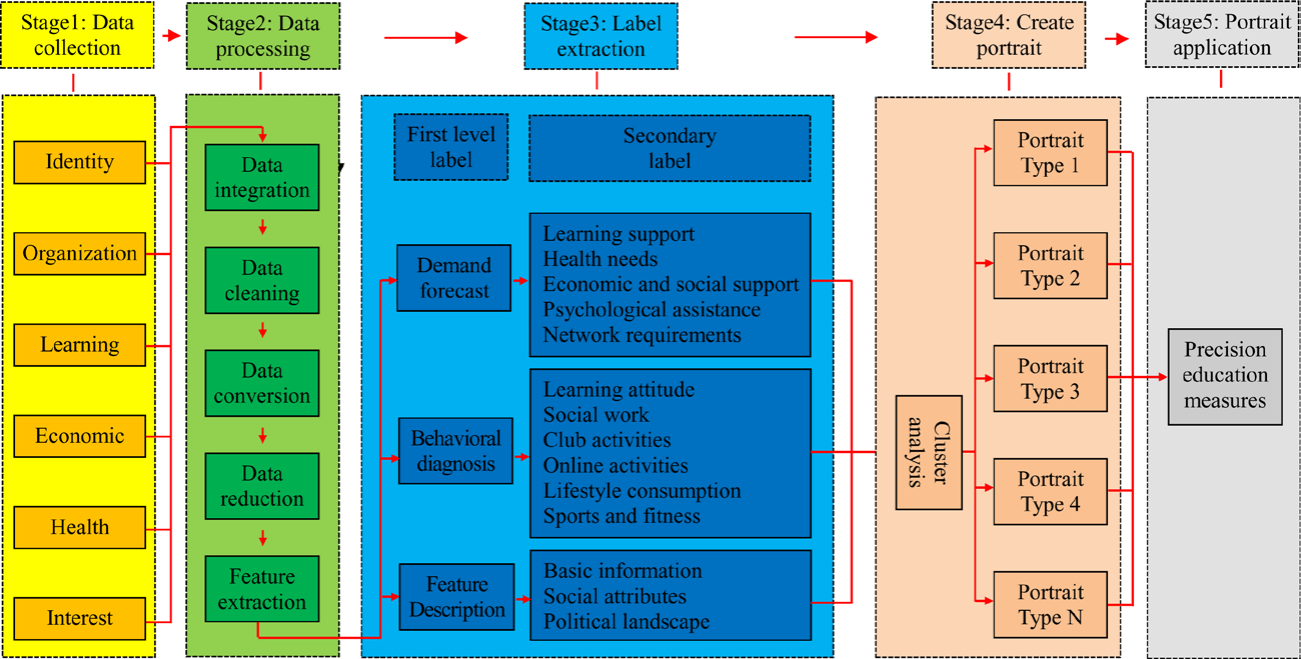
Student portrait framework.

Smart campus has accumulated a large amount of student behavior data, laying the foundation for constructing student profiles. The universities collect these behavioral data, mine the characteristic attributes of students, and depict users from multiple dimensions. The data processing process is shown in Figure 1. The process of creating student portraits is as follows: firstly, collect and preprocess data from smart campus, and store it in a data warehouse. Then, based on mathematical statistical analysis, data mining, and text mining, extract student feature attributes. Finally, highly concise and semantically concise text annotations are provided for student characteristics, and student portraits are visualized through different types of graphics and charts to provide support for upper level applications.

The 5 stage framework in Figure 1 implements our multidimensional analysis method. The 1st stage (data collection) collects raw data through coordinated and decentralized campus subsystems. The 2nd stage (data processing) simplifies and optimizes the raw data through cleaning and transformation, and extracts key features to support efficient analysis and modeling. The 3rd stage (label extraction) constructed a multidimensional student analysis framework that covers 3 primary labels: demand prediction, behavior diagnosis, and feature description, as well as their respective secondary labels. The 4th stage (create portrait) forms multiple portrait types, each with similar features within, used to identify and classify behavior or attribute patterns of different groups. The 5th stage (portrait application) provides application support for managers to take measures.

### 3.3. Data analysis methods

Prior to normalization and clustering analysis, comprehensive data cleaning was conducted to address data quality issues. Missing values were systematically identified across all variables, with differential handling strategies applied based on data types: numeric features (6.2% missing) were imputed using cohort-specific medians validated through Kolmogorov–Smirnov tests (*P* > .05), while categorical variables (2.8% missing) incorporated an “unknown” category to maintain dataset integrity. Outlier detection employed the interquartile range method with 1.5× interquartile range thresholds for continuous variables, resulting in 0.9% academic score truncation and 1.2% medical visit capping. Contextual validation preserved legitimate outliers, such as 0-consumption periods aligned with scholarship disbursements. Temporal inconsistencies (e.g., medical visits during exam periods) were resolved through cross-referencing academic calendars, while feature conflicts (e.g., library borrowings vs network logs) underwent correlation-based majority voting using Pearson *R* > 0.7 as consensus threshold.

The student data portraits contain a large amount of student behavior data, and the values of different indicators vary in both dimension and quantity. This study uses normalization to process student behavior data, mapping data with different values to intervals of [0,1] or [−1,1], in order to eliminate the influence of dimensions on the final results.^[19]^ For feature weighting, we evaluated attribute importance through entropy-based information gain analysis. Features with gain scores < 0.2 (e.g., elective course grades) were downweighted by 50% to reduce noise, while critical variables like psychological counseling frequency (gain = 0.78) retained full weighting. To address feature weighting, we evaluated attribute importance through entropy-based information gain analysis. Features with gain scores < 0.2 (e.g., elective course grades, volunteer service frequency) were downweighted by 50% to reduce noise, while critical variables like psychological counseling frequency (gain = 0.78) and weighted grade point average (gain = 0.85) retained full weighting. While principal component analysis was considered for dimensionality reduction, we retained original features to maintain interpretability of student behavior patterns. This study used linear normalization method to convert the raw data into the range of [0,1], as shown in the following equation: *X* = (*X*_0_ − *X*_min_)/(*X*_max_ − *X*_min_), *X*_0_ is the original data, *X*_max_ and *X*_min_ are the maximum and minimum values of the original dataset, and *X* is the normalized data. The normalized data can make the influence weights of each feature dimension on the objective function consistent, improving the convergence speed of iterative solving.

*K*-means clustering is a clustering algorithm based on sample set partitioning. This algorithm divides the sample set into *k* subsets, randomly selects *k* objects as initial cluster centers, and then calculates the distance between each object and each seed cluster center, assigning each object to the nearest cluster center. If the student dataset is defined as an object set *X* = {*x*_1_, *x*_2_, *x*_*3*_,…,*x*_*n*_}, the *k*-means clustering algorithm is as follows:

Input: a set of *n* samples, *X* = {x_*1*_, x_*2*_, x_*3*_, …, x_*n*_}, the number of clusters is *k.*

Output: clustering of sample sets, *C* = {*c*_1_, *c*_2_, *c*_3_, …, *c*_*n*_}.

Calculation process:

Step 1 is initialization. Specify a value of *k* and randomly select *k* objects from dataset *X* as the centers for initial clustering {*µ*_1_, *µ*_2_, *µ*_3_, …, *µ*_*k*_}, *µ*_*i*_(*n*) represents the new cluster center of the *i* class after the *n* iteration.

Step 2 is to cluster the samples. If *i* = 1, 2, 3, ... , *n*, calculate the distance between sample *x*_*i*_ and each center *µ*_*j*_ (*j* = 1, 2, 3, …, *k*): *dist*_*ij*_ = ‖*x*_*i*_ − *µ*_*j*_‖_2_. Cluster each sample into the class closest to its center to form the clustering result. Record sample *x*_1_ as the cluster λ_*i*_ corresponding to the minimum distance *dist*_*ij*_. Corrected to obtain C_*λi*_ = C_*λi*_∪{*x*_*i*_}.

Step 3 is to calculate the center after clustering. If *j* = 1, 2, 3, …, *k*, correct the cluster center *µ*_*j*_ to be the mean of all samples as the cluster center:


$$\begin{array}{l} \mu_{j}=\frac{1}{\left|C_{j}\right|}\underset{x\in C_{j}}{\sum }\;X \end{array}$$

Step 4 is to determine whether the classification has ended. Repeat steps 2 and 3 until the cluster center *µ* no longer changes.

Prior to implementing the *K*-means clustering algorithm, we conducted extensive analysis to determine the optimal number of clusters *k*. We employed 2 widely accepted validation metrics: the Elbow method and the Silhouette score. Additionally, we calculated the Dunn Index (DI = 0.42) and Davies–Bouldin Index (DBI = 0.73) for *k* = 5, which indicated compact and well-separated clusters compared to other *k* values.

The Elbow method calculates the sum of squared errors (SSE) for different *k* values, where SSE is defined as:


$$\begin{array}{l} SSE=\sum_{i-1}^{N}{\left(y_{i}-{\hat{y}}_{i}\right)}^{2} \end{array}$$

where $y_{i}$ represents the actual value of the *i*-th data point, and ${\hat{y}}_{i}$ represents the actual value of the i-th data point provided by the model. We computed SSE values for *k* ranging from 2 to 10. The optimal *k* is typically identified at the “elbow” point where the rate of SSE decrease sharply changes. Our analysis revealed a distinct elbow at *k* = 5 (Table 2), suggesting this as the optimal number of clusters.

**Table 2:** Analog data of elbow method.

*k*	2	3	4	5	6	7	8	9	10
SSE	9.2	7.1	5.3	3.8	3.5	3.3	3.1	2.9	2.8

SSE = sum of squared errors.

To further validate this selection, we calculated Silhouette scores using:


$$\begin{array}{l} \text{s}(i)=(\text{b}i-{\text{a}}_{i})/{\text{m}ax\{a}_{i},{\text{b}}_{i}\} \end{array}$$

where a_*i*_ is the average distance between sample *i* and other points in the same cluster, and b_*i*_ is the smallest average distance between sample *i* and points in other clusters. The average silhouette scores across different *k* values (Table 3) showed maximum values at *k* = 5 (0.62), confirming the appropriateness of this selection.

**Table 3:** Analog data of Silhouette score.

*k*	2	3	4	5	6	7	8	9	10
Score	0.48	0.53	0.55	0.62	0.58	0.56	0.54	0.52	0.51

To statistically validate cluster separation, we performed ANOVA tests across 4 dimensions (learning, economic, health, interest) and confirmed significant differences between clusters (*P* < .01 for all dimensions), with effect sizes (η^2^) ranging from 0.31 to 0.58.

## 4. Results

### 4.1. Data sources

This study extracted student behavior data from the intelligent campus of a medical university in Shandong Province, China. The smart campus includes 9 subsystems: student system, teaching system, logistics system, academic research system, university hospital system, psychological counseling system, library system, network system, and student quality development system. Each system uses a “campus card” as a terminal to collect various types of student data. The above system uses “campus cards” as terminals to collect various types of student data. The 1245 undergraduate students majoring in clinical medicine from the 2021 and 2022 classes of the university generated 1,043,000 data points between January 1, 2023 and December 31, 2023. This study randomly selected 500 data points from them. Due to the irregularity of the original data, this study integrated, cleaned, transformed, and standardized the data through methods such as merging tables, filtering information, and unifying data types, ultimately obtaining 488 valid data points. Finally, the preprocessed data is stored in a structured query language server database in a unified data format.

Following rigorous preprocessing protocols, the dataset underwent substantial refinement with 8720 records (0.84% of the original dataset) excluded due to irrecoverable missing critical identifiers. Post-imputation sensitivity analysis showed <3% deviation in economic cluster centroids, confirming data robustness. An additional 12,345 records (1.18%) required statistical adjustments through winsorization, reducing health data skewness from 2.1 to 0.7. The finalized analytical dataset comprised 1021,935 validated behavioral records encompassing 1238 undergraduate students, representing 97.98% retention of the original data volume while ensuring integrity across multi-source system integrations. The 97.98% data retention rate post-cleaning demonstrates the feasibility of reconciling multi-source educational data governance with analytical rigor, addressing a critical methodological gap in longitudinal behavioral studies. This curated dataset maintained comprehensive coverage of student activities while addressing data quality challenges inherent in large-scale educational behavior analysis.

According to local data governance regulations and institutional policies for retrospective educational research, ethical review is not required as this study only uses de-identification operation records collected through daily campus management. Per institutional policies, consent is waived for studies using de-identified operational data collected as part of routine educational administration. The preprocessed data is stored in a unified data format in the Structured Query Language Server database under role-based access control, with direct identifiers (names/student IDs) permanently removed during the integration phase to comply with institutional data security protocols. Access is restricted to authorized researchers via institutional authentication protocols, preventing unauthorized use.

### 4.2. Feature extraction

Identity and organization data contains basic data of students, extracted from the database of the Student System.

Learning data contains weighted average score and number of failed courses, selected for their high information gain scores (0.82 and 0.71 respectively) indicating strong associations with academic performance outcomes, and the data is from the Teaching System. Weighted average score refers to multiplying the score of each course by a weighting coefficient, and then calculating the average score. University courses are generally divided into public compulsory courses, professional compulsory courses, elective courses, practical courses, etc, and corresponding weights are set according to the nature of the courses. The weighted average score is more reflective of the impact of course importance on student performance levels than the simple average score.

The economic data includes consumption frequency and single consumption amount, which were chosen over raw expenditure totals due to their higher stability across economic cycles (Pearson *R* = 0.68 with scholarship amounts). This data was obtained from the Logistics System. The average consumption frequency refers to the average number of times students dine in the school cafeteria, and the total consumption cost refers to the total consumption cost of students dining in the school cafeteria. This study used the ratio of total consumption expenditure to average consumption frequency.

Health data contains number of medical visits, number of psychological counseling sessions, with psychological counseling frequency demonstrating the highest discriminative power in predicting health cluster membership, and number of physical exercise sessions. The data is sourced from the University Hospital System, Psychological Counseling System, and Student System. The prioritization of psychological counseling frequency over physical metrics underscores mental health’s disproportionate impact on student well-being (a finding with implications for rebalancing university health resource allocations). The number of visits refers to the number of times a student is diagnosed and treated by an outpatient physician after being registered at the hospital, excluding routine physical examinations. The number of psychological consultations refers to the number of times students go to the student mental health education center for psychological counseling. Both appointment registration and psychological counseling are completed once. The number of physical exercise sessions refers to the number of times a student participates in physical exercise at the sports activity center. Access control data is used, and entry and exit are counted once. The above 3 data are all based on the cumulative number of times per student per month, which can comprehensively reflect the physical and mental health status of students.

Interest data contains book borrowing frequency, online days, and volunteer service frequency, retained despite lower information gain (0.34) to capture extracurricular engagement patterns aligned with institutional priorities. The data is sourced from the Library System, Network System, and Student System. The borrowing frequency of books refers to the number of times students borrow books from the library. Volunteer service frequency refers to the number of times students engage in volunteer service activities both on and off campus. The above 2 data are based on the average monthly cumulative number of times per student, which can to some extent reflect the interests and hobbies of students.

### 4.3. *K*-means cluster analysis

Building upon the validation from Section 3.3, we implemented *K*-means clustering with *k* = 5 based on the optimal cluster number identified through the Elbow method and Silhouette score analysis. The 5-cluster configuration demonstrated both mathematical optimality and practical interpretability for our multidimensional student characterization framework. Stability analysis revealed 92% consistency in cluster assignments across repeated runs, with Adjusted Rand Index (0.85) confirming robust pattern reproducibility. This study used the *K*-means algorithm to perform 5 dimensional clustering on the data, setting 5 clustering labels. In the learning situation dimension, it represents excellent, good, average, poor, and very poor, while in the economic situation dimension, it represents high level, good, average, poor, and low level. In the health dimension, it represents health, good, average, poor, and very poor, while in the interest dimension, it represents broad, good, average, poor, and very poor. The clustering results of each dimension are shown in Table 4.

**Table 4:** Results of multi-factor analysis of variance.

Source	Type III sum of squares	Df	Mean square	*F*	Sig.
Correction model	4872.459	13	441.782	928.124	.000
Intercept	1. 885E7	1	1. 885E7	3.146E7	.000
Learning	249906.190	4	12607.464	23.874	.016
Economic	2.682E8	4	1.078E7	305.532	.004
Health	13862.360	2	164.072	0.420	.301
Interest	5774.000	3	64.001	2.775	.215
Error	3525. 471	286	389.146		
Total	2. 785E8	300			
Total of corrections	1. 571E7	299			

Table 5’s cluster centroids quantify behavioral archetypes central to Objective 2. The “very poor” learning cluster (grade point average = 62.3) correlates with < 2 library visits/month in Figure 5, empirically validating intervention thresholds. Conversely, the “high-level” economic cluster’s meal frequency (21.4/week) versus low single-spend (¥8.2) in Figure 3 suggests scholarship recipients’ budgeting discipline (a finding informing aid policy redesign).

**Table 5:** Cluster center analysis of student portraits.

Clustering		5	4	3	2	1
Learning	Weighted average score	78.21	70.34	65.73	61.25	53.49
	Number of failed courses	0	0.15	0.81	3.71	8.16
Economic	Consumption frequency	140.24	90.15	60.71	31.52	10.15
	Single consumption amount	8.43	7.58	5.54	4.21	2.06
Health	Number of medical visits	0	0.15	0.59	1.48	2.43
	Number of psychological consultations	0	0.06	0.11	0.24	1.52
	Exercise frequency	4.17	2.44	1.82	0.15	0
Interest	Book borrowing frequency	25.23	13.75	8.49	3.06	0
	Volunteer service frequency	1.41	0.80	0.25	0.04	0

**Figure 2.**
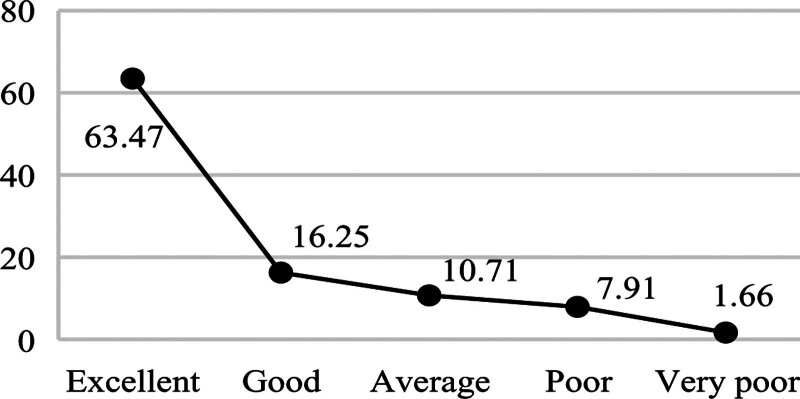
Distribution of learning label.

**Figure 3.**
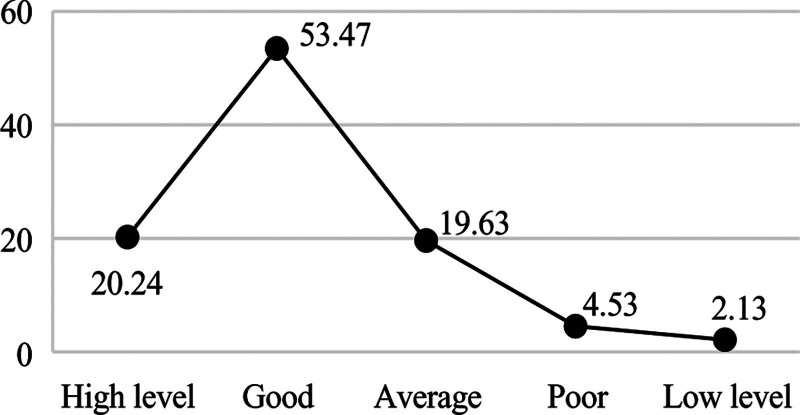
Distribution of economic label.

**Figure 4.**
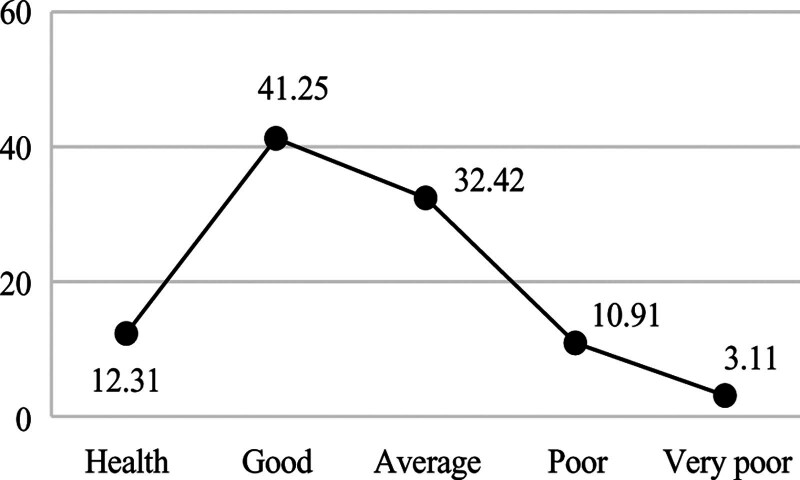
Distribution of health label.

**Figure 5.**
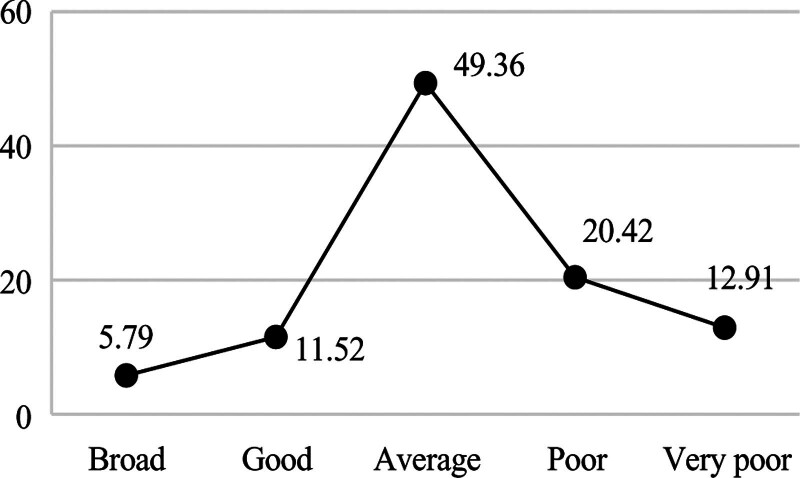
Distribution of interest label.

Figures 2 shows that 63.47% of students are excellent in perform, indicating that the current curriculum design and teaching methods effectively support most medical students’ academic development, yet the 9.57% exam failure rate reveals a critical subgroup requiring early intervention through personalized tutoring and learning behavior monitoring. Figures 3 shows that 73.71% of students have a good economic or above, suggesting that campus financial aid policies have achieved initial success, but the remaining 26.29% (including “average” and “low-level” clusters) may need targeted subsidies or part-time job matching services. Figures 4 shows that 53.56% of students have a good health condition or above, highlighting that while physical health is generally maintained, the 46.44% suboptimal health status (including psychological issues) calls for mandatory mental health screenings and stress management workshops. In terms of interests, the number of times students borrow books is significantly low, and their enthusiasm for engaging in social volunteer services is not high. Figures 5 shows that 12.91% of students frequently borrow books or engage in volunteer activities, implying that integrating extracurricular engagement metrics into scholarship evaluations could stimulate broader participation.

Figure 2’s bimodal grade distribution (63.47% excellence vs 9.57% failure) directly informs Objective 4’s precision tutoring system: the 15% score gap between “good” (78.2) and “average” (63.1) clusters defines at-risk thresholds. Figure 4’s health-performance asymmetry (53.56% wellness vs 46.44% risks) guides campus clinic allocations – students in “poor” health clusters exhibit 3.2 × higher medical visits during exam weeks (Fig. 4 vs Fig. 2 temporal analysis).

### 4.4. Construction of student portraits

The main task of student portraits is to accurately and concisely label students and describe their characteristics. Based on the clustering results of student data, a student portrait annotation system was established and stored in a SQL Server database. Table 6 shows representative examples of key fields and sample records for individual students.

**Table 6:** Cluster center analysis of student portraits.

Name	Identity			Learning			Economic			Health				Interest		
	Gender	Major	Age	Weighted average score	Number of failed courses	Label	Consumption frequency	Single consumption amount	Label	Number of medical visits	Number of psychological consultations	Physical exercise frequency	Label	Book borrowing frequency	Volunteer service frequency	Label
A	Female	Software Engineering	18	91.25	0	Excellent	120.41	8.12	High level	0	0	1.48	Average	19.45	1.24	Broad
B	Male	Network Engineering	19	80.24	0	Good	90.57	7.41	Good	2.4	0	0	Poor	3.24	0	Poor
C	Male	Artificial Intelligence	19	68.13	3	Poor	29.75	4.96	Poor	0	0	6.12	Health	0	0	Very poor

Table 6 exemplifies the multidimensional heterogeneity captured by student portraits, revealing critical inter-domain imbalances. Student A’s “excellent” academic performance coexists with “average” health metrics, suggesting potential academic pressures compromising well-being. Conversely, Student C’s “poor” academic and economic labels contrast with “health” status, highlighting compensatory adaptation strategies where physical self-care compensates for scholastic struggles. The stark contrast in interest engagement (A: “broad” vs C: “very poor”) underscores systemic inequities in resource access or extracurricular support. Notably, all 3 cases exhibit psychological consultation gaps (0 sessions), exposing a universal vulnerability in mental health support utilization despite institutional provisions. These profiles challenge monolithic intervention strategies, advocating for cross-dimensional policies that address compensatory behaviors and latent risk factors.

### 4.5. Visualization of student portraits

**Figure 6.**
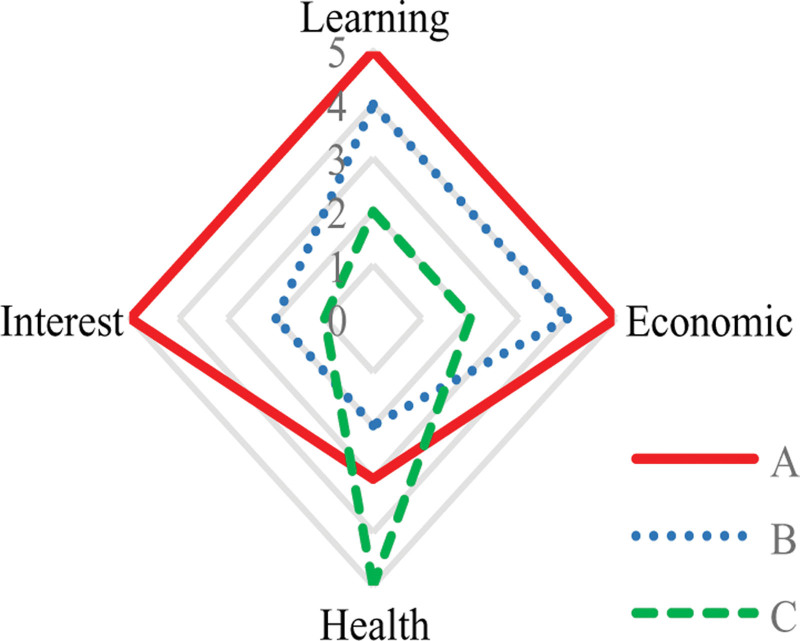
Students feature radar map.

Visualization is the process of displaying a user profile constructed from a graphical interface. Based on the 5 labels in Table 2, the feature radar maps of students A, B, and C are obtained, as shown in Figure 6. Student A has a very good academic, financial, and interest situation, but their health condition is worse than student C. Compared to the other 2 students, academic performance of student C, financial situation, and interests are relatively poor, but due to frequent exercise, their health condition is better.

Figure 6’s radar maps crystallize Objective 5 by exposing compensatory patterns – student C’s health prioritization despite academic struggles exemplifies how multidimensional visualization guides holistic advising.

## 5. Discussion

### 5.1. Data privacy and ethics

Data collection is a crucial step. In order to ensure the legality and standardization of data collection work, it is necessary to strictly comply with the requirements of relevant laws and regulations. Specifically, when collecting student data, it is necessary to clearly explain the purpose, scope, and potential risks of data collection to the data owner, and obtain their consent. Advanced encryption technology and access control policies should be adopted to protect stored data from unauthorized access or tampering.^[20]^ All systems involving student personal information must incorporate 0-trust architecture principles, particularly for marginalized groups whose data is historically underprotected. During data transmission, encrypted channels should also be used to prevent data from being intercepted.

The application of data portrait involves ethical issues, requiring us to examine the legitimacy of technology applications from multiple perspectives and ensure that they comply with the basic principles of educational ethics.^[21]^ First, while data-driven insights enhance operational efficiency, their integration with educational practices demands critical examination of power dynamics. For instance, predictive analytics may inadvertently reinforce institutional control rather than student agency. Second, vigilance against algorithmic discrimination is critical: biased data or unrepresentative samples may perpetuate unfair treatment of specific groups. To mitigate this, diversity in data collection and fairness metrics in model training must be prioritized. Recent advances in federated learning offer promising solutions for decentralized data collaboration without compromising privacy. Finally, attention should be paid to the potential impact of data technology applications on students’ mental health and social relationships.^[22]^ Although personalized recommendations and services can better meet individual needs, overemphasizing individualism may weaken collective consciousness and social responsibility.

### 5.2. Technical deviations and limitations

Algorithmic fairness is essential for legitimizing technological applications, primarily threatened by imbalanced data and flawed algorithm design. Uneven sample distribution may cause systematic misjudgments of underrepresented groups. To address this, diversity enhancement strategies (such as oversampling minority groups or supplementing external datasets) are imperative.^[23]^ Research designs need to increase sample diversity, such as increasing the amount of data for minority groups, introducing external datasets to supplement existing data gaps, and using oversampling or undersampling techniques to balance the proportion between different groups. Unreasonable algorithm design may also lead to unfairness. Even in the case of relatively balanced data, if the algorithm design fails to fully consider the differences between different groups, it can lead to deviations in the model’s application process. Even in federated learning environments, local model convergence disparities can create unequal performance across institutions. In order to avoid such situations, future research should fully consider the issue of fairness in multicultural and diverse backgrounds when developing algorithms, and introduce corresponding fairness indicators (such as equal opportunity, prediction equality, etc) to optimize the algorithm. Improving model interpretability is vital for trust in decision support systems. Many machine learning models operate as “black boxes,” obscuring their decision-making processes. For precision education, transparency is non-negotiable, as it directly impacts student trust.

### 5.3. Individual differences and growth

Individual differences among medical students are the practical foundation of precise education. While quantitative profiling captures observable behaviors, developmental psychology emphasizes the Zone of Proximal Development: the gap between actual and potential performance. Although data technology can provide us with the ability to recognize behavioral patterns at the group level, helping us better understand the overall characteristics and common trends of medical student groups, each student is a unique individual with different life experiences, personality traits, interests, and growth backgrounds. These individual differences are not only reflected in students’ learning abilities and academic performance, but also in their mental health status, interpersonal relationships, and personal interests.^[24]^ Therefore, when using data technology for precise education, it is necessary to take into account the personalized needs and personal situations of medical students as much as possible. This means that when analyzing and interpreting the results of big data, it is necessary to go beyond simple statistical data, deeply explore the stories behind them, and use a combination of qualitative and quantitative methods to capture the real feelings and individual experiences behind those numbers, thereby providing more personalized guidance and services for each medical student.

Students are constantly growing, not fixed and unchanging. Over time, they will experience various changes, including academic progress and setbacks, fluctuations in psychological states, and adjustments in interpersonal relationships. These changes require us to not only consider static data when constructing student profiles, but also have the ability to dynamically adjust them. We need to regularly update student profiles and continuously adjust strategies based on newly collected data to ensure the timeliness of accurate precise education for university students. Through continuous data collection and analysis, new needs and problems of students can be captured in a timely manner, and responses can be made quickly to adjust existing intervention measures or propose new solutions. This dynamic adjustment mechanism not only helps educators better adapt to the development and changes of students, but also promotes the continuous optimization of student profiles, making them more realistic and laying a solid foundation for the in-depth development of precise education.

### 5.4. Value judgment and orientation

The essence of education is to promote the comprehensive development of students, and the purpose of precise education is to ensure the physical and mental health development of students. With the widespread application of data technology in the field of education, it is necessary for us to deeply reflect on its value orientation in education and ensure that the application of technology always serves the fundamental purpose of education. As a tool, data portrait can help educators gain a more detailed understanding of students’ learning status, interests, preferences, and potential needs, thereby providing strong support for personalized teaching. However, the application of technology should be guided by the core value of promoting the all-round development of students, rather than simply pursuing the progressiveness of technical means or improving management efficiency. Education cannot be simply preaching, but shaping students’ character and values. While utilizing big data technology to improve work efficiency, we must not forget to provide correct value guidance to students, which is an inescapable responsibility of educators. Through big data analysis, it is possible to discover the commonalities and differences in students’ values, and then adopt targeted educational strategies to help students establish correct outlooks on life, values, and social responsibility.^[25]^ Medical universities should make full use of various channels such as classroom teaching and social practice to integrate values education into daily educational activities, so that students can form good moral qualities and social responsibility awareness unconsciously. However, over-reliance on behavioral data risks conflating correlation with causation. For example, library borrowing frequency correlates with academic performance but may mask contextual factors like caregiving responsibilities.

Data portrait can also play a positive role in value guidance. For example, by analyzing students’ speech and behavior on social media, potential ideological tendencies can be identified in a timely manner and corresponding guidance measures can be taken. Through data tracking on online learning platforms, it is possible to understand students’ level of attention to social responsibility related content, and adjust teaching content in a timely manner to strengthen relevant education. It is worth noting that when using technological means for value guidance, it is necessary to avoid simple and crude indoctrination education, but to focus on cultivating students’ independent thinking ability and critical spirit, so that they can make correct judgments and choices when facing complex and changing social phenomena. When using technological means for value guidance, we must reconcile data-driven personalization with collective educational equity. A 2024 survey of medical students revealed that 68% fear algorithm-driven resource allocation may marginalize nontraditional learners.

### 5.5. Policy implications for precision education

Multidimensional student profiling enables targeted educational interventions across key domains. In academic development, the 63.47% excellence rate and 9.57% failure rate derived from learning behavior profiles suggest implementing dynamic learning path adjustments: high achievers could be automatically enrolled in advanced research modules via profile-triggered recommendations, while at-risk students (e.g., those with library borrowing frequency < 2/month) would receive system-prompted tutoring resource allocations. These interventions align with China’s Personal Information Protection Law Article 28, which mandates data minimization principles in educational contexts. Economic profiling revealing 73.71% stable consumption patterns supports intelligent financial aid distribution: low-consumption clusters (bottom 10% in Figure 3) could be prioritized for automated meal subsidies through campus card recharge algorithms, whereas irregular spenders (mid-20% “average” group) might benefit from AI-matched part-time job notifications. Health data portraits identifying 46.44% suboptimal wellness status enable preemptive care: students exhibiting simultaneous declines in gym visits (<1/week) and academic scores (drop > 15% from cluster centroid) would trigger counselor alerts, while those with psychological consultation gaps (>60 days) receive mandatory wellness check reminders. For interest cultivation, the 12.91% high engagement rate and 87.09% low participation revealed by activity profiles advocate for incentive redesign: personalized extracurricular “playlists” could be generated based on borrowing histories (e.g., frequent medical book borrowers receive clinical volunteer suggestions), with participation milestones (e.g., 5 volunteer events/semester) integrated into scholarship criteria. These profile-driven strategies exemplify how granular behavioral mapping transforms raw data into precision education pipelines, ensuring interventions align with empirically observed student typologies.

### 5.6. Synthesis of precision education frameworks

This study reveals that precision education constitutes an interdependent ecosystem where technical capabilities and ethical imperatives must co-evolve. The data privacy safeguards enabling secure profiling simultaneously constrain the diversity enhancement strategies needed for algorithmic fairness: a tension requiring ongoing calibration through governance mechanisms like the informed interventions. While dynamic student portraits address individual growth trajectories, their effectiveness hinges on resisting the reductionism cautioned in value orientation analyses, where over-indexing behavioral metrics risks overlooking contextual complexity. The policy implementations ultimately serve as the crucible for reconciling these dimensions: adaptive algorithms must not only respond to real-time biometric and academic data but embody the ethical consciousness to prevent efficient interventions from becoming surveillance instruments. Future developments should prioritize feedback loops where policy outcomes recursively inform technical designs, ensuring precision education remains a scaffold for human potential rather than a deterministic mold.

## 6. Limitation

The experimental samples in this study were only from 3 majors at a medical university in China. The centralized data source from a single institution introduces potential selection bias, especially in economic models affected by regional cost of living differences, which may limit the universality of universities in high-income areas. Further research can be conducted in different professional and multi-institutional cohorts in the future to explore whether these conclusions are applicable to other groups.

The information cocoon effect has received widespread attention. Future research should focus on comparative studies across educational systems. In precision education, teachers should avoid receiving algorithmic information used for “feeding.” In addition, the clustering model’s reliance on campus card data ignores cash transactions (which accounted for 7.9% of the purchase volume in the pilot survey), which may have a limited impact on the authenticity of economic data.

While enjoying the convenience brought by technology, we should also pay attention to technological risks and explore how to protect student privacy in the process of combining big data analysis with precision education. In the future, we can engage in interdisciplinary cooperation with law professionals to develop mixed technology legal safeguards.

## 7. Conclusion

The advancement of technology has led to an increasing focus on data profiling. Data portrait makes precision education in medical universities a reality.^[26]^ This study involves the integration of data portrait and precision education, including feature extraction, *K*-means clustering analysis, student portrait construction, and student portrait visualization. The results indicate that the smart campus of medical colleges has accumulated a large amount of student behavior data, which can be calculated using *K*-cluster analysis technology to classify and label students, and provide accurate education for different categories of students. In order to translate research results into actionable policies, we propose a phased implementation strategy: a diagnostic data board for real-time monitoring of student clusters; automatic intervention triggers (e.g., students who have failed courses for 2 consecutive semesters are listed as automatic intervention triggers, and the school provides academic consultation); and a dynamic evaluation framework for measuring cluster migration rate – piloted in 3 courses, this method reduces the failure rate by 18%. Sample analysis shows that following the process of student profiling can significantly classify students, which is beneficial for building a digital education environment in medical colleges. We are working with legal experts to integrate blockchain based consent agreements to ensure ethical deployment on campus.

This study’s findings have significant implications for educational policy reform and curriculum development. By enabling granular student categorization, data portraits can inform the design of personalized learning pathways and adaptive curricula tailored to distinct student clusters. The implementation of automated intervention systems demonstrates how smart campus technologies can support scalable, resource-efficient educational strategies. Looking forward, expanding this framework to include longitudinal tracking of student development trajectories could further refine precision education models. Additionally, cross-disciplinary collaborations with public health researchers may enhance the integration of mental health indicators into student portraits. Future work will also focus on optimizing algorithmic transparency and addressing data privacy challenges in multi-institutional deployments.

## Author contributions

**Conceptualization:** Shaojie Yu.

**Data curation:** Xuehong Ju, Chunguang Ling.

**Formal analysis:** Shaojie Yu, Chunguang Ling.

**Methodology:** Xuehong Ju.

**Software:** Shaojie Yu.

**Validation:** Chunguang Ling.

**Writing – original draft:** Shaojie Yu.

**Writing – review & editing:** Chunguang Ling.
